# The Role of the Social Environment on Adaptive Neuroplasticity in Early Development

**DOI:** 10.1155/2019/5375849

**Published:** 2019-10-08

**Authors:** A. Guzzetta, L. Murray, R. Montirosso, P. F. Ferrari

**Affiliations:** ^1^Department of Clinical and Experimental Medicine, University of Pisa, Italy; ^2^IRCCS Stella Maris Foundation, Pisa, Italy; ^3^School of Psychology and Clinical Language Sciences, University of Reading, Reading, UK; ^4^0-3 Centre for the At-Risk Infant, IRCCS “E. Medea”, Bosisio Parini, Lecco, Italy; ^5^CNRS/Université Claude Bernard, Institut des Sciences Cognitives Marc Jeannerod, Lyon, France

Adaptive neuroplasticity is the ability of the brain to reorganize neural connectivity in response to experience. It is potentially more efficient during early development, allowing children to be more fast-paced, for example, in learning a new language or in achieving new, complex, motor skills required, for example, for proficient playing of musical instruments. At the same time, deprivation or perturbation of experiences during early life can impact the development of functions relating to the nature of the deprivation (e.g., see [1]).

In this very critical phase for infant neurodevelopment, social interactions, such as in the caregiver-infant dyad, seem to play a particularly important role in modulating infant experiences. The objective of this special issue was, therefore, to examine the scientific evidence for cerebral plasticity in relation to social interactions with the environment during early development, focusing on risk factors for atypical neurodevelopment and on the role of specific sensory, motor, and social experiences.

The topics covered by this special issue were the objectives of a very stimulating workshop held in Erice, Sicily, and hosted at the Ettore Majorana Foundation and Centre for Scientific Culture in October 2017. This special issue represents the fruitful results of the scientific exchanges and stimulating ideas circulating among scholars and participants during the meeting.

The fundamental role of early social experience was addressed by C. A. Nelson III et al. by reviewing current knowledge concerning the effects of psychosocial deprivation in rodents, nonhuman primates, and institutionalized infants. More specifically, the authors summarized the results of two randomized controlled trials from institutionalized children in Romania who were followed up after adoption out of the institutional setting into families. They discussed how deprivation can have a range of short- and long-term psychodevelopmental and behavioral consequences, with clear differences in brain structure, and changes in cellular and molecular level markers according to the timing and duration of institutional deprivation.

Even when interactions between the caregiver and infant are possible from the start of the postnatal period, there are nevertheless several factors that can interfere and negatively affect newborn development. One of them is the presence of perinatal adverse events, putting the infant at risk of neurodevelopmental disorders. F. Festante et al. reviewed the studies reporting on the quality of early parent-infant interactions in dyads where the infant was at high risk of neuromotor disorders, such as cerebral palsy, showing that both infant and maternal behaviors within early interactions are markedly compromised. Abnormalities in infant behavior tended to persist beyond the first semester of life, with infants being less engaged in the interaction, while early intrusive maternal behaviors later evolved into more controlling and attention-directing behaviors. The role of mother-infant interactions in populations at risk was indirectly confirmed by the study of G. Sgandurra et al. who showed how an early intervention that involves parents, as in the CareToy model, seems to be effective in reducing maternal distress, as compared to standard care for preterm infants. This is consistent with the findings of C. Vandormael et al. who reviewed current knowledge on the effects of preterm birth on the development of language skills, showing how environmental and neurophysiological factors can influence preterm neuroplasticity with potential short- and long-term effects on language development.

The ability of the infant to express social signals seems particularly relevant in the development of the dyadic interaction. By studying infants with cleft lip, in comparison to typical infants, L. Murray et al. showed, through microanalytic video analysis, a lower rate of maternal mirroring of infant expressions by mothers of infants with cleft lip. Interestingly, this was associated with reduced maternal gaze to the infant's mouth, suggesting a high sensitivity of parent-infant interactions to specific variations in interactants' appearance and behavior. Some of the consequences of infant impaired ability to express emotions due to facial palsy were explored by Y. Nicolini et al. who tested the hypothesis that, in children with Moebius syndrome, the impaired motor expression of a given emotion is directly linked to the autonomic responses associated with that same emotion. By means of functional infrared thermal imaging, they were able to demonstrate how the impairment of facial movement attenuates the intensity of emotional experience, probably due to the weakening of action-perception cortical mechanisms and the diminished activation of autonomic responses typically associated with the facial expression of emotions. The role of empathy during development is confirmed by the study from A. Milone et al. who explored the relationship between empathy and callous-unemotional traits in children with a diagnosis of conduct disorders, showing a significant correlation of callous-unemotional traits with both cognitive and emotional dimensions of empathy.

As one of the objectives of this special issue was to expand our understanding of the role of specific sensory experiences on early neuroplasticity, we welcomed contributions exploring the mechanisms of sensory processing or the effects of early sensory deprivation. In a review paper, O. Chorna et al. examined the recent literature on fetal and neonatal processing of a complex, but specific, sensory stimulation, that is, music. In particular, the authors examined the behavioral, neurophysiological, and neuroimaging literature describing fetal and neonatal music perception and processing, concluding with recommendations for music stimulation within the framework of early socioemotional development. New evidence of the effects of early auditory deprivation and visual deprivation on neural processing and function was explored in two separate studies. J. Andin et al. reported fMRI evidence of different patterns of activation of parietal numerical processing regions in adult deaf singers as compared to typically hearing adult controls, shedding light on the possible underpinnings of the poorer numerical abilities reported in congenitally deaf individuals. Z. Zhou et al. reported effects of early visual deprivation on the microstructure and functional brain connectivity in early blind adolescents compared to normal-sighted controls, thus providing new insights into the mechanisms underlying the neural reorganization of the brain in adolescents with early visual deprivation.

Expanding our knowledge of the role of the social environment on adaptive neuroplasticity in early development in a multilayer perspective (i.e., from biomarkers to behavior) can support collaborations between basic researchers and clinical contexts. Indeed, improved understanding of some of the mechanisms involved provides potential directions for promoting the translation of the principles of neuroplasticity into implementation for more effective early interventions, in order to reduce the impact of neurodevelopmental disability across a range of clinical conditions.
